# Impact of C-reactive protein-albumin-lymphocyte index as a prognostic marker for the patients with undergoing gastric cancer surgery

**DOI:** 10.3389/fnut.2025.1556062

**Published:** 2025-03-12

**Authors:** Makoto Toda, Hiroaki Musha, Takefumi Suzuki, Takashi Nomura, Fuyuhiko Motoi

**Affiliations:** ^1^Department of Surgery, Yamagata Prefectural Central Hospital, Yamagata, Japan; ^2^First Department of Surgery, Yamagata University, Yamagata, Japan

**Keywords:** C-reactive protein-albumin-lymphocyte index, prognostic biomarker, gastric cancer, postoperative complications, nutrition

## Abstract

**Introduction:**

The significance of the C-reactive protein-albumin-lymphocyte index [CALLY index (CI)] as a prognostic factor in gastric cancer remains unexplored. Therefore, this study assessed the utility of the CI as a predictor of short-term postoperative outcomes and long-term prognosis after gastric cancer surgery.

**Methods:**

This study consisted of two cohorts. Cohort 1 included 120 patients who underwent distal gastrectomy for clinical stages I–III primary gastric cancer between November 2022 and March 2024. Patients were categorized into high- and low-CI groups, and complications were classified accordingly. Propensity score matching was performed based on clinical stage, surgical approach, and lymph node dissection extent, yielding 40 matched cases. The relationship between preoperative CI and short-term postoperative outcomes was analyzed. Cohort 2 included 358 patients with pathological stages I–III gastric cancer who underwent distal gastrectomy between January 2014 and December 2017. Preoperative CI was assessed, and its association with long-term outcomes was evaluated. Prognostic factors were also analyzed.

**Results:**

In Cohort 1, the preoperative CI was associated with short-term postoperative outcomes. Compared with the high-CI group, the low-CI group developed significantly more complications, including postoperative pneumonia. In Cohort 2, the 5-year overall survival (OS) and recurrence-free survival (RFS) differed significantly between the high and low CI groups. CI was an independent prognostic factor for OS and RFS.

**Conclusion:**

The CI reflects patients' overall systemic conditions and may be a valuable predictor of short-term outcomes and long-term prognosis following gastric cancer surgery.

## 1 Introduction

Gastric cancer is the fifth most common type of cancers worldwide (1). Despite recent advances in early detection, endoscopy, surgery, and chemotherapy, the prognosis of patients with gastric cancer remains poor and continues to be a significant global health concern (2). Patients with gastric cancer often experience malnutrition and poor nutritional status, which influence the occurrence of perioperative complications and prognosis in patients undergoing surgery (3, 4). Therefore, the development of a simple and accurate nutritional assessment index may help to identify high-risk patients, improve prognosis, and treatment strategies.

Several inflammation/nutrition-related indices, including serum C-reactive protein (CRP) levels (5), serum albumin levels (6), neutrophil-to-lymphocyte ratio (NLR) (7), platelet-to-lymphocyte ratio (PLR) (8), and Controlling Nutritional Status (CONUT) score (9), have been used to examine the relationship between prognosis and inflammation/nutrition status. CRP-albumin-lymphocyte index [CALLY index (CI)] was recently proposed by Iida et al., as a new inflammation/nutrition-related index (10). The CI considers three factors: serum albumin level, lymphocyte count, and CRP as indicators of nutritional, immune, and inflammatory statuses, respectively. This index may better reflect the nutritional status, inflammatory response, and immune system function. Although the relationships between CI and short- and long-term prognoses have been reported for various types of cancer, few studies have specifically focused on gastric cancer (11, 12). CI may play a pivotal role in reducing perioperative complication rates and improving prognosis by identifying patients who require interventions and facilitate appropriate management.

This study investigated the relationship between the CI and short-term outcomes as well as long-term prognosis, to predict outcome in patients undergoing gastrectomy.

## 2 Materials and methods

### 2.1 Ethics

The protocol for this research project was approved by a suitably constituted Ethics Committee of the institution and it conforms to the provisions of the Declaration of Helsinki and the 2017 Ethical Guidelines for Medical Research Involving Human Subjects Ethics Committee of Yamagata Prefectural Central Hospital (YPCH) (approval No. 22-117). All Cohort 1 participants provided informed consent as appropriate. For Cohort 2 participants, an opt-out method was used to obtain consent, and the requirement of written informed consent was waived.

### 2.2 Recruitment

#### 2.2.1 Cohort 1

**Figure 1:**
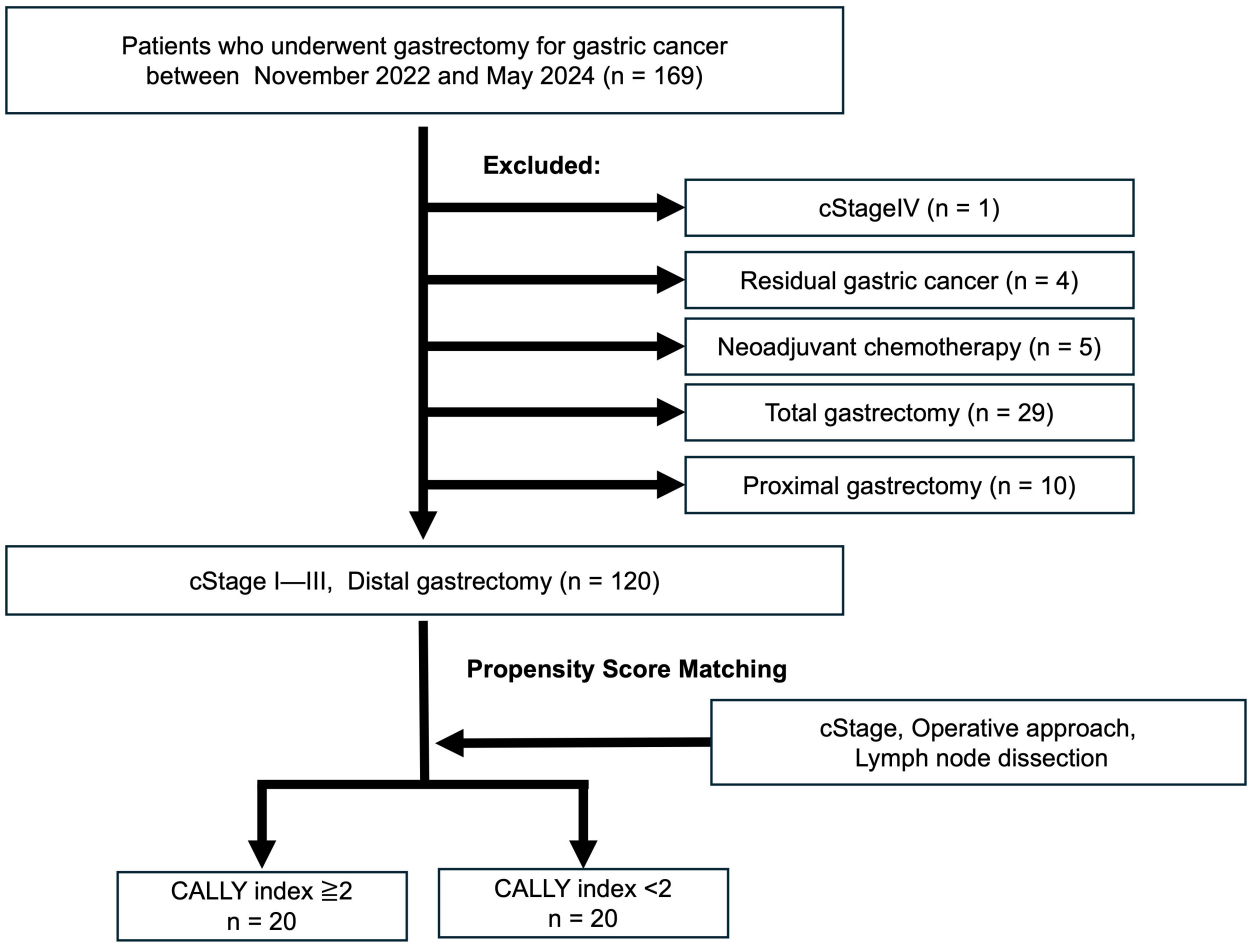
Cohort 1 study population. A total of 120 patients who underwent distal gastrectomy for clinical stage (cStage) I–III gastric cancer between November 2022 and March 2024 were divided into high and low CALLY groups based on a cutoff value of 2. Propensity score matching was performed to adjust for clinical stage, surgical approach, and extent of lymph node dissection, resulting in 20 patients each in the high and low CALLY groups. CALLY, C-reactive protein-albumin-lymphocyte.

This cohort was evaluated for short-term outcomes and included 169 patients who underwent gastrectomy for primary gastric cancer at YPCH between November 2022 and March 2024. The patients with (1) with clinical stage (cStage) IV disease (*n* = 1), (2) residual gastric cancer (*n* = 4), (3) treated with neoadjuvant chemotherapy (*n* = 5), which was excluded due to its potential impact on nutritional status and inflammatory response, and (4) who did not undergo distal gastrectomy (*n* = 39), were excluded from this study. Finally, prospectively collected data on consecutive 120 patients who underwent distal gastrectomy for cStages I–III disease were eligible for analysis (Figure 1).

#### 2.2.2 Cohort 2

**Figure 2:**
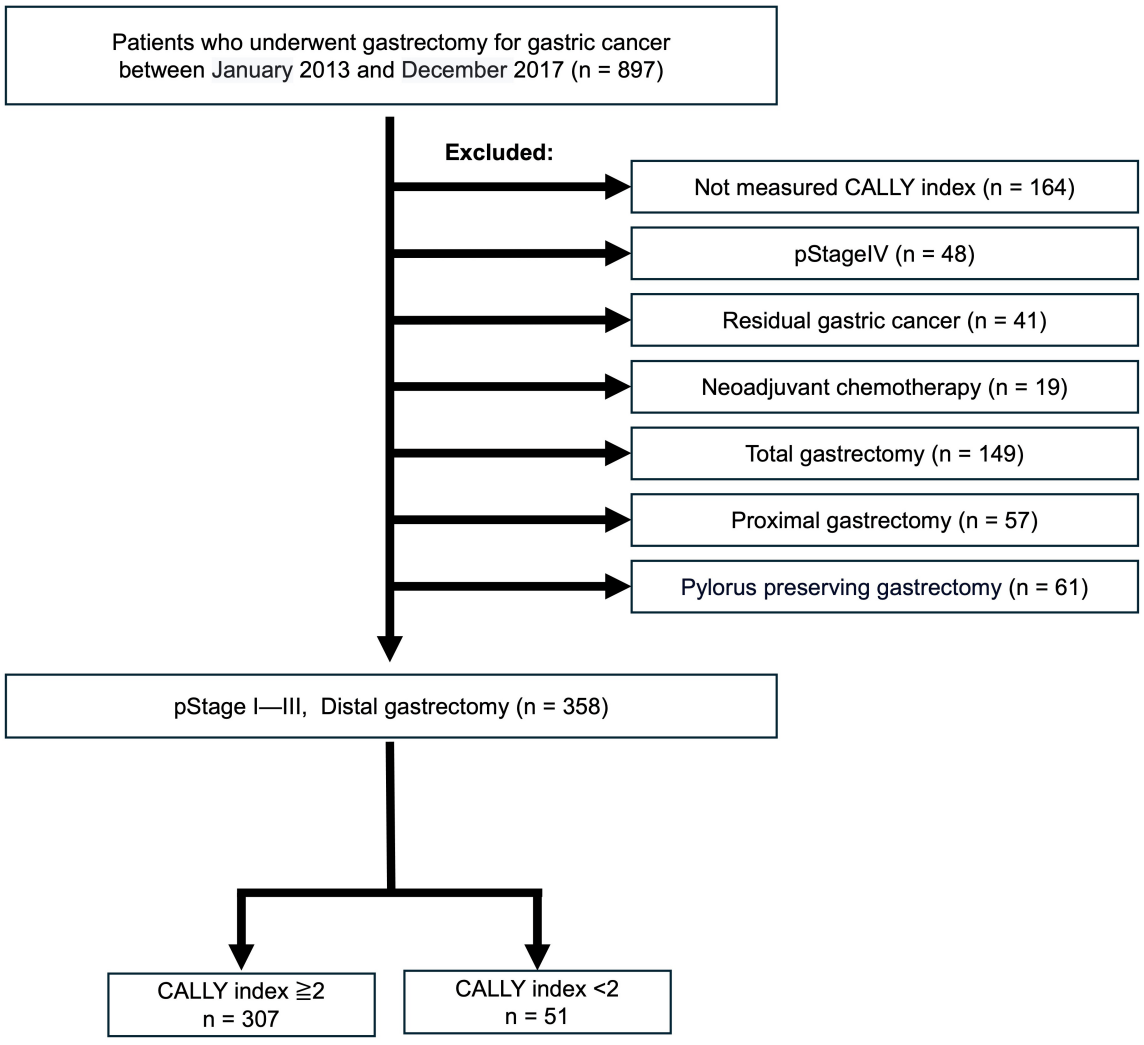
Cohort 2 study population. Out of 897 patients with primary gastric cancer treated between January 2014 and December 2017, 358 patients with pathological stage (pStage) I–III disease who underwent distal gastrectomy and had preoperative CALLY measurements were selected. These patients were divided into high and low CALLY groups based on a cutoff value of 2. CALLY, C-reactive protein-albumin-lymphocyte.

This cohort was evaluated for long-term outcomes and included 897 patients with primary gastric cancer who underwent gastrectomy at YPCH between January 2013 and December 2017. The patients with (1) no preoperative CI data (*n* = 164), (2) cStage IV disease (*n* = 48), (3) residual gastric cancer (*n* = 41), (4) treated with neoadjuvant chemotherapy (*n* = 19), which was excluded due to its potential impact on nutritional status and inflammatory response, and (5) that did not undergo distal gastrectomy (*n* = 267) were excluded. Retrospectively collected data on consecutive 358 patients with pathological stages (pStages) I—III disease who underwent distal gastrectomy were eligible for analysis (Figure 2).

In Cohort 1, routine blood parameters, including lymphocyte count, serum albumin level, serum CRP level, serum carcinoembryonic antigen (CEA) level, and serum carbohydrate antigen 19-9 (CA19-9) level were collected. The following clinicopathologic variables, surgical outcomes, and postoperative course were collected from the medical records and databases: age, sex, body mass index (BMI), Charlson comorbidity index, clinical stage, histologic type, depth of tumor invasion, extent of lymph node involvement, surgical approach, and lymph node dissection. In Cohort 2, in addition to the variables collected on Cohort 1, information on the administration of adjuvant chemotherapy was obtained, and the date and cause of death or the last follow-up date was recorded. Overall survival (OS) was defined as the time from the date of surgery to the date of death from any cause or the last follow-up. Similarly, recurrence-free survival (RFS) was defined as the time from the date of surgery to the date of confirmed recurrence, death, or the last follow-up. Additionally, disease-specific survival (DSS) was analyzed. Clinicopathologic evaluations for all patients were conducted according to the Japanese Classification of Gastric Cancer (13). Postoperative complications were evaluated according to the Clavien-Dindo (C-D) classification of the Japan Clinical Oncology Group (JCOG) (14). The CI was calculated as albumin (g/dL) × lymphocyte count (/μL)/(CRP (mg/dL) × 10^4^). All variables used in this study were complete, and no missing data were observed. Therefore, no imputation or additional handling for missing data was necessary.

### 2.3 Statistics

The cutoff value for the CALLY Index (CI) was determined to be 2 based on the receiver operating characteristic (ROC) curve analysis, using the highest Youden Index to optimize the balance between sensitivity and specificity. The analysis yielded an area under the curve (AUC) of 0.60. Patients were then divided into high- and low-CI groups according to this cutoff value. Propensity score matching was conducted in Cohort 1 to minimize potential confounding factors, and standardized mean differences (SMDs) were calculated post-matching to assess the balance of baseline characteristics between the two groups. Statistical analyses were performed using JMP Pro version 16. χ2 and Wilcoxon tests were used to compare the two groups. Survival curves were generated using the Kaplan–Meier method, and the log-rank test was used to compare survival between groups. The Cox proportional hazards model was used to assess the prognostic factors. Univariate and multivariate analyses were performed to identify factors influencing survival. In the multivariate analysis, factors that demonstrated significant differences in the univariate analysis were included for evaluation. Statistical significance was set at *p* < 0.05.

## 3 Results

### 3.1 Cohort 1

Patient backgrounds are summarized in Table 1. Seventy-six patients were male, with a median age of 73 years. A total of 98 patients had CI ≥ 2, whereas 22 patients had CI < 2. Sixty-one and 59 patients had early and advanced gastric cancer, respectively. Clinical staging revealed 71, 14, and 35 patients with cStage I, II, and III disease, respectively. Forty-three patients underwent open surgery, and 77 patients underwent minimally invasive surgery. Among the patients that underwent minimally invasive surgery, 40 and 37 underwent laparoscopic and robot-assisted procedures, respectively.

**Table 1:** Characteristics of patients in cohort 1.

	**Overall cohort**				**After matching**			
**Variable**	**Total (*****n*** = **120)**	**CALLY index**		***p*** **-value**	**Total (*****n*** = **40)**	**CALLY index**		***p*** **-value**	**SMD**
		≧**2 (*****n*** = **98)**	<**2 (*****n*** = **22)**			≧**2 (*****n*** = **20)**	<**2 (*****n*** = **20)**		
Sex, male/female	76/44	64/34	12/10	0.34	26/14	14/6	12/8	0.51	0.21
Age, years, median (range)	73 (26–91)	73 (26–90)	77 (45–91)	0.067	75 (45–90)	75 (51–90)	76 (45–88)	0.81	0.062
Body mass index, kg/m^2^, median (range)	22.2 (14.7–36.5)	22.6 (14.7–36.4)	20.4 (17.6–32.1)	0.077	22.1 (16.3–32.1)	23.8 (16.3–26.0)	20.4 (17.6–32.1)	0.023	0.55
Charlson comorbidity index, median (range)	4 (0–7)	4 (0–7)	5 (3–7)	0.12	5 (2–7)	5 (2–7)	5 (3–7)	0.75	0.18
Serum albumin, g/dL, median (range)	4.2 (1.9–5.0)	4.3 (3.1–5.0)	2.9 (1.9–4.4)	0.0002	3.9 (1.9–5.0)	4.0 (3.4–5.0)	2.9 (1.9–4.4)	0.0089	1.5
Serum C-reactive protein, mg/dL, median (range)	0.06 (0.01–8.95)	0.05 (0.01–0.29)	0.69 (0.19–8.95)	< 0.0001	0.20 (0.01–8.95)	0.05 (0.01–0.27)	0.61 (0.19–8.95)	< 0.0001	0.84
Lymphocyte count/μL, median (range)	1,600 (500–3,100)	1,600 (800–3,100)	1,300 (500–2,800)	0.042	1,500 (500–3,100)	1,600 (1,000–3,100)	1,300 (500–2,800)	0.23	0.34
Serum CA19-9, U/mL, median (range)	6.9 (2.1–1,568.7)	7.1 (2.1–994.5)	6.2 (2.1–1,568.7)	0.98	7.6 (2.1–1,568.7)	7.8 (2.1–889.8)	4.4 (2.1–1,568.7)	0.69	0.05
Serum CEA, ng/mL, median (range)	2.5 (1.7–178.7)	2.5 (1.7–178.7)	2.6 (1.7–34.1)	0.91	2.7 (1.7–34.1)	2.9 (1.7–11.9)	2.7 (1.7–34.1)	0.63	0.20
Histological type, difuse type/intestinal type	81/39	68/30	13/9	0.35	26/14	15/5	11/9	0.18	0.43
Clinical T factor, 1/2/3/4	61/16/19/24	56/14/15/13	5/2/4/11	0.002	8/5/6/21	3/3/4/10	5/2/2/11	0.70	0.08
Clinical N factor, 0/1/2/3	81/14/24/1	73/9/15/1	8/5/9/0	0.0051	15/7/17/1	7/3/9/1	8/4/8/0	0.74	0.10
Clinical stage, I/II/III	71/14/35	66/11/21	5/3/14	0.002	10/6/24	5/3/12	5/3/12	1.00	0.00
Surgical approach, open/MIS	43/77	26/72	17/5	< 0.0001	30/10	15/5	15/5	1.00	0.00
Lymph node dissection, D1+/D2	64/56	58/40	6/16	0.01	8/32	4/16	4/16	1.00	0.00

CALLY, C-reactive protein-albumin-lymphocyte; CA19-9, carbohydrate antigen 19-9; CEA, carcinoembryonic antigen; MIS, minimally invasive surgery; SMD, standardized difference.

Sex, age, and BMI did not differ significantly between the high- and low-CI groups. The factors related to CI in the low-CI group included significantly lower serum albumin levels (4.3 vs. 2.9 g/dL, *p* = 0.002), higher serum CRP levels (0.05 vs. 0.69 mg/dL, *p* < 0.0001), and lower lymphocyte counts (1,600 vs. 1,300/μL, *p* = 0.042). cStage was significantly more advanced in patients with lower CI (*p* = 0.002). The surgical approach also differed significantly, with more patients undergoing open surgery (*p* < 0.001) and D2 lymph node dissection (*p* = 0.0067). Since open surgery and extensive lymph node dissection are believed to strongly influence the postoperative course, propensity score matching was performed to adjust for clinical stage, surgical approach, and extent of lymph node dissection (Figure 1). After propensity score matching, low-CI (*n* = 20) and high-CI (*n* = 20) were selected. Most variables showed standardized mean differences (SMDs) below 0.1 after matching; however, gender (SMD = 0.21), BMI (SMD = 0.55), and histological type (SMD = 0.43) remained imbalanced. The N factor showed an SMD of 0.10, indicating a minimal imbalance. Meanwhile, clinical stage, surgical approach, and extent of lymph node dissection achieved perfect balance with an SMD of 0.00 (Table 1).

Surgical outcomes are shown in Table 2. Overall, the median operating time was 274 min, median blood loss was 84 mL, postoperative hospital stay was 10 days, and postoperative complications occurred in 14 patients (35.0%). The operative time and blood loss did not differ significantly between the high- and low-CI groups; however, the low-CI group had a significantly longer postoperative hospital stay (9 vs. 12 days, *p* = 0.027) and a significantly higher incidence of ostoperative complications (15.0% vs. 55.0%, *p* < 0.0001). Postoperative complications included pancreatic fistula (six patients, 15.0%), intra-abdominal abscess and postoperative pneumonia (five patients, 12.5%), and anastomotic leakage (three patients, 7.5%). Although the incidence of pancreatic fistula, intra-abdominal abscess, and anastomotic leakage did not differ significantly between the high- and low-CI groups, the incidence of postoperative pneumonia was significantly higher in the low-CI group (0 vs. 25.0%, *p* = 0.017).

**Table 2:** Postoperative outcomes in cohort 1.

**Variables**	**Total (*n* = 40)**	**CALLY index**		***p*-value**
		≧**2 (*****n*** = **20)**	<**2 (*****n*** = **20)**	
Operative time, min, median (range)	274 (103–463)	250 (207–424)	287 (103–463)	0.32
Blood loss, mL, median (range)	84 (0–1,317)	64 (0–1,317)	109 (0–790)	0.44
Postoperative hospital stay, days, median (range)	10 (7–70)	9 (7–70)	12 (7–70)	0.027
Postoperative complication (Clavien-Dindo grade ≧II)	14 (35.0%)	3 (15.0%)	11 (55.0%)	0.008
**Details of complications**				
Pancreatic fistula	6 (15.0%)	2 (10.0%)	4 (20.0%)	0.38
Intra-abdominal abscess	5 (12.5%)	2 (10.0%)	3 (15.0%)	0.63
Postoperative pneumonia	5 (12.5%)	0 (0.0%)	5 (25.0%)	0.017
Anastomotic leakage	3 (7.5%)	2 (10.0%)	1 (5.0%)	0.55
Others	2	0	2	

CALLY, C-reactive protein-albumin-lymphocyte.

### 3.2 Cohort 2

Patient backgrounds are summarized in Table 3. Two hundred and twenty-nine patients were male, with a median age of 70 years. A total of 307 patients had CI ≥ 2, whereas 51 patients had CI < 2. Open surgery was performed in 207 patients, whereas minimally invasive surgery (MIS), all of which was laparoscopic, was performed in 151 patients. Among these patients, 215, 82, and 61 were classified as pStage I, pStage II, pStage III disease, respectively. Venous and lymphatic invasions were observed in 311 and 111 patients, respectively. Postoperative complications were observed in 86 (24.0%) patients. Adjuvant chemotherapy was discussed with the pStage II/III patients and administered to those who opted for it (90 patients).

**Table 3:** Characteristics of patients in cohort 2.

**Variables**	**Total (*n* = 358)**	**CALLY index**		***p*-value**
		≧**2 (*****n*** = **307)**	<**2 (*****n*** = **51)**	
Sex, male/female	227/131	193/114	34/17	0.60
Age, years, median (range)	70 (35–92)	62 (35–91)	75 (54–92)	< 0.0001
Body mass index, kg/m^2^, median (range)	22.8 (13.9–34.7)	23.2 (16.1–34.7)	21.7 (13.9–30.4)	0.015
Charlson comorbidity index, median (range)	4 (0–9)	4 (0–9)	4 (0–8)	0.0014
Serum albumin, g/dL, median (range)	4.3 (1.9–5.3)	4.3 (2.3–5.3)	3.5 (1.9–4.8)	< 0.0001
Serum C-reactive protein, mg/dL, median (range)	0.049 (0.01–27.1)	0.039 (0.01–0.8)	1.0 (0.25–2.71)	< 0.0001
Lymphocyte count,/μL, median (range)	1,700 (500–4,100)	1,700 (700–4,100)	1,400 (500–3,900)	0.0004
Serum CA19-9, U/mL, median (range)	5.6 (1.9–18,812)	5.3 (1.9–1,539.1)	6.5 (1.9–18,812)	0.038
Serum CEA, ng/mL, median (range)	2.3 (0.5–238.0)	2.2 (0.5–238.0)	3.2 (1.0–74.9)	0.0005
Histological type, difuse type/intestinal type	229/129	198/115	37/14	0.17
Surgical approach, open/MIS	207/151	167/140	40/11	0.0013
Lymph node dissection, D1+/D2	137/221	119/188	18/33	0.64
Operative time, min, median (range)	286 (178–717)	285 (208–644)	281 (178–717)	0.48
Blood loss, mL, median (range)	57 (0–2,523)	57 (0–2,523)	89 (0–1,630)	0.33
Pathological T factor, 1/2/3/4	198/64/28/68	179/50/23/55	19/14/5/13	0.043
Pathological N factor, 0/1/2/3	232/57/41/28	199/48/35/25	33/9/6/3	0.94
Pathological stage, I/II/III	215/82/61	192/63/52	23/19/9	0.023
Venous invasion, yes/no	311/41	270/37	41/10	0.14
Lymphatic invasion, yes/no	247/111	216/91	31/20	0.17
Postoperative complication (Clavien-Dindo grade ≧II)	86 (24.0%)	57 (18.6%)	29 (56.9%)	< 0.0001
Postoperative hospital stay, days, median (range)	9 (6–145)	8 (6–145)	12 (7–82)	< 0.0001
Adjuvant chemotherapy, yes/no	90/268	80/227	10/41	0.33
Adjuvant chemotherapy (pathological stage II,III), yes/no	90/54	80/36	10/18	0.0012

CALLY, C-reactive protein-albumin-lymphocyte; CEA, carcinoembryonic antigen; CA19-9, carbohydrate antigen 19-9; MIS, minimally invasive surgery.

No significant differences in sex were observed between the high- and low-CI groups. The low-CI group was significantly older (62 vs. 75 years, *p* < 0.0001) and had higher serum CEA levels (2.2 vs. 3.2 ng/mL, *p* = 0.0005), higher serum CRP levels (0.039 vs. 1.0 mg/dL, *p* < 0.0001), and lower lymphocyte counts (1,700 vs. 1,400/μL, *p* = 0.0004). In the low-CI group, 40 cases underwent open surgery and 11 cases underwent MIS, whereas in the high-CI group, 167 cases underwent open surgery and 119 cases underwent MIS, showing a significant difference (*p* = 0.00113). However, there were no significant differences in operative time or blood loss. The low-CI group had a higher incidence of perioperative complications (18.6 vs. 56.9%, *p* < 0.0001) and a longer postoperative hospital stay (8 vs. 12 days, *p* < 0.0001). The high-CI group had higher rates of pStage I disease than the low-CI group (*p* = 0.023). Venous and lymphatic invasion did not differ significantly between the groups. In patients with pStages II/III, adjuvant chemotherapy was administered significantly less frequently in the low-CI group (68.7 vs. 35.7%, *p* = 0.012).

**Figure 3:**
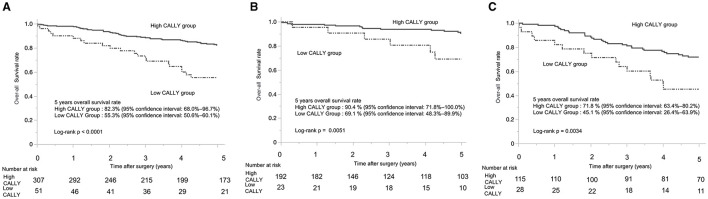
Kaplan-Meier curves of overall survival in patients with gastric cancer who underwent distal gastrectomy. **(A)** Kaplan-Meier curves of overall survival for patients with stage I–III gastric cancer who underwent distal gastrectomy. **(B)** Kaplan-Meier curves of overall survival for patients with pathological stage I gastric cancer who underwent distal gastrectomy. **(C)** Kaplan-Meier curves of overall survival for patients with pathological stage II/III gastric cancer who underwent distal gastrectomy. CALLY, C-reactive protein-albumin-lymphocyte.

The 5-year OS rate in Cohort 2 was 78.2% (95% confidence interval: 73.5–82.9%). When stratified by CI, the low-CI group had a significantly worse 5-year OS rate than the high-CI group (55.3%, 95% confidence interval: 50.6–60.1% vs. 82.3, 95% confidence interval: 68.0–96.7%; *p* < 0.0001) (Figure 3A). The 5-year OS rate for the patients with pStage I was 87.9% (95% confidence interval: 64.1–100.0%), whereas that for patients with pStage II/III was 66.5% (95% confidence interval: 58.6–74.5%). Among the patients with pStage I, those in the low-CI group had a significantly poorer 5-year OS than those in the high-CI group (69.1%, 95% confidence interval: 48.3–89.9% vs. 90.5%, 95% confidence interval: 71.8–100.0%; *p* = 0.0051) (Figure 3B). Similarly, in patients with pStages II/III, the low-CI group showed a significantly worse 5-year OS rate than the high-CI group (45.1%, 95% confidence interval: 26.4–63.9% vs. 71.8%, 95% confidence interval: 63.4–80.2%; *p* = 0.0034) (Figure 3C).

**Figure 4:**
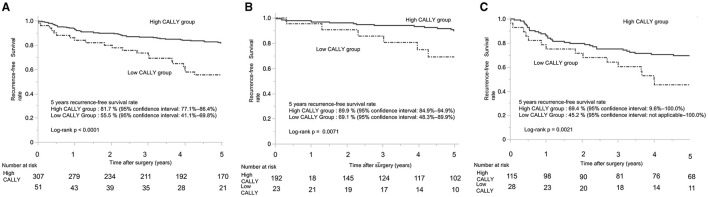
Kaplan-Meier curves of recurrence-free survival in patients with gastric cancer who underwent distal gastrectomy. **(A)** Kaplan-Meier curves of recurrence-free survival for patients with stage I–III gastric cancer who underwent distal gastrectomy. **(B)** Kaplan-Meier curves of recurrence-free survival for patients with pathological stage I gastric cancer who underwent distal gastrectomy. **(C)** Kaplan-Meier curves of recurrence-free survival for patients with pathological stage II/III gastric cancer who underwent distal gastrectomy. CALLY, C-reactive protein-albumin-lymphocyte.

The 5-year RFS rate showed a significant difference between the CI groups. For patients with pStages I-III, the low-CI group had a markedly lower 5-year RFS rate compared to the high-CI group (55.5%, 95% confidence interval: 41.1–69.8% vs. 81.7, 95% confidence interval: 77.1–86.4%; *p* < 0.0001) (Figure 4A). Among patients with pStage I, the low-CI group exhibited a significantly lower 5-year RFS rate compared to the high-CI group (69.1%, 95% confidence interval: 48.3–89.9% vs. 89.9%, 95% confidence interval: 84.9–94.9%; *p* = 0.0071) (Figure 4B). Similarly, in patients with pStages II/III, the low-CI group demonstrated a significantly poorer 5-year RFS rate compared to the high-CI group (45.2%, 95% confidence interval: not applicable−100.0 vs. 69.5%, 95% confidence interval: 9.6–100.0%; *p* = 0.021) (Figure 4C). In contrast, the 5-year DSS rate for patients with pStages I-III showed no significant difference between the groups, with the DSS rates in the low-CI and high-CI groups being nearly identical (91.2%, 95% confidence interval: 84.9–92.9%, vs. 89.3%, 95% confidence interval: 81.5–100.0%, *p* = 0.61).

Univariate and multivariate analyses were conducted to identify factors influencing 5-year OS and RFS. The evaluated factors included sex, age, BMI, surgical approach, operative time, blood loss, pStage, venous invasion, lymphatic invasion, postoperative complications, adjuvant chemotherapy, and CI.

In the univariate analysis for 5-year OS, significant differences were observed for age [hazard ratio (HR): 2.23, 95% confidence interval: 1.34–3.71, *p* = 0.0022]; surgical approach (HR: 3.96, 95% confidence interval: 2.02–7.76, *p* < 0.0001); pStage (HR: 3.20, 95% confidence interval: 1.89–5.40, *p* < 0.0001); venous invasion (HR: 3.10, 95% confidence interval: 1.83–5.25, *p* < 0.0001); lymphatic invasion (HR: 2.48, 95% confidence interval: 1.53–4.03, *p* = 0.0002); postoperative complications (HR: 2.00, 95% CI: 1.12–3.30, *p* = 0.0061); and CI (HR: 3.07, 95% confidence interval: 1.83–5.16, *p* < 0.0001). In the multivariate analysis, significant factors included age (HR: 1.79, 95% confidence interval: 1.05–3.01, *p* = 0.030); surgical approach (HR: 2.17, 95% confidence interval: 1.04–4.55, *p* = 0.033); venous invasion (HR: 1.87, 95% confidence interval: 1.05–3.33, *p* = 0.034); and CI (HR: 2.11, 95% confidence interval: 1.22–3.63, *p* = 0.007). CI had the highest hazard ratio among all evaluated factors (Table 4).

**Table 4:** Univariate and multivariate analysis of overall survival in cohort 2.

**Variables**		**Univariate analysis**				**Multivariate analysis**	
		**HR**	**95% CI**	***p*** **-value**	**HR**	**95% CI**	***p*** **-value**
Sex	Male/female	1.57	0.91–2.71	0.10			
Age	≧70/ < 70 years	2.23	1.34–3.71	0.0022	1.79	1.05–3.01	0.030
BMI	< 22.7 /≧22.7 kg/m^2^	1.41	0.87–2.28	0.16			
Surgical approach	Open/MIS	3.96	2.02–7.76	< 0.0001	2.17	1.04–4.55	0.038
Operative time	< 286/≧286 min	1.52	0.90–2.55	0.11			
Blood loss	≧57/ < 57 mL	1.34	0.82–2.18	0.25			
Pathological stage	II, III/I	3.20	1.89–5.40	< 0.0001	1.60	0.84–3.05	0.15
Venous invasion	Yes/no	3.10	1.83–5.25	< 0.0001	1.87	1.05–3.33	0.034
Lymphatic invasion	Yes/no	2.48	1.53–4.03	0.0002	1.35	0.77–2.35	0.29
Postoperative complication (C-D grade)	≧grade II/grade 0,I	2.00	1.123.30	0.0061	1.80	0.82–2.32	0.23
Adjuvant chemotherapy	Yes/no	1.66	1.02–2.73	0.43			
CALLY index	< 2/≧2	3.07	1.83–5.16	< 0.0001	2.11	1.22–3.63	0.007

HR, hazard ratio; CI, confidence interval; BMI, body mass index; CA19-9, carbohydrate antigen 19-9; CEA, carcinoembryonic antigen; MIS, minimally invasive surgery; C-D, Clavien-Dindo; CALLY, C-reactive protein-albumin-lymphocyte.

For 5-year RFS, univariate analysis revealed significant differences for age (HR: 2.26, 95% confidence interval: 1.37–3.72, *p* = 0.0014); surgical approach (HR: 4.01, 95% confidence interval: 2.11–7.63, *p* < 0.0001); pStage (HR: 3.56, 95% confidence interval: 2.13–5.94, *p* < 0.0001); venous invasion (HR: 3.05, 95% confidence interval: 1.82–5.12, *p* < 0.0001); lymphatic invasion (HR: 2.54, 95% confidence interval: 1.59–4.07, *p* < 0.0001); postoperative complications (HR: 2.13, 95% confidence interval: 1.31–3.45, *p* = 0.002); adjuvant chemotherapy (HR: 2.15, 95% CI: 1.34–3.46, *p* = 0.0015); and CI (HR: 2.77, 95% confidence interval: 1.66–4.62, *p* < 0.0001). In the multivariate analysis for 5-year RFS, significant factors were age (HR: 2.02, 95% confidence interval: 1.18–3.46, *p* = 0.011); venous invasion (HR: 1.82, 95% confidence interval: 1.03–3.23, *p* = 0.041); and CI (HR: 1.81, 95% confidence interval: 1.05–3.10, *p* = 0.032) (Table 5). CI was an independent prognostic factor for OS and RFS.

**Table 5:** Univariate and multivariate analysis of recurrence-free survival in cohort 2.

**Variables**		**Univariate analysis**		**Multivariate analysis**			
		**HR**	**95% CI**	***p*** **-value**	**HR**	**95% CI**	***p*** **-value**
Sex	Male/female	1.63	0.96–2.75	0.07			
Age	≧70/ < 70 years	2.26	1.37–3.72	0.0014	2.02	1.18–3.46	0.011
BMI	< 22.7/≧22.7 kg/m^2^	1.46	0.91–2.34	0.11			
Surgical approach	Open/MIS	4.01	2.11–7.63	< 0.0001	2.02	0.99–4.11	0.053
Operative time	< 286/≧286 min	1.37	0.83–2.552	0.21			
Blood loss	≧57/ < 57 mL	1.32	0.82–2.12	0.25			
Pathological stage	II, III/I	3.56	2.13–5.94	< 0.0001	1.51	0.72–3.13	0.27
Venous invasion	Yes/no	3.05	1.82–5.12	< 0.0001	1.82	1.03–3.23	0.041
Lymphatic invasion	Yes/no	2.54	1.59–4.07	< 0.0001	1.31	0.76–2.25	0.33
Postoperative complication (C-D grade)	≧grade II/grade 0,I	2.13	1.31–3.45	0.002	1.44	0.87–2.39	0.16
Adjuvant chemotherapy	Yes/no	2.15	1.34–3.46	0.0015	1.46	0.79–2.68	0.22
CALLY index	< 2/≧2	2.77	1.66–4.62	< 0.0001	1.81	1.05–3.10	0.032

HR, hazard ratio; CI, confidence interval; BMI, body mass index; CA19-9, carbohydrate antigen 19-9; CEA, carcinoembryonic antigen; MIS, minimally invasive surgery; C-D, Clavien-Dindo; CALLY, C-reactive protein-albumin-lymphocyte.

## 4 Discussion

The results of this study demonstrated that a CI < 2 was significantly associated with postoperative complications and worst overall survival in patients undergoing distal gastrectomy. To our knowledge, this is the first comprehensive evaluation of preoperative CI, highlighting its dual predictive value for short- and long-term outcomes in resected gastric cancer. In general, poor nutritional status is a serious problem that adversely affects patient quality of life and condition, leading to increased morbidity, length of hospitalization, mortality, and healthcare costs (15). Nutritional status is a critical factor for the treatment of various carcinomas. Poor nutritional status adversely affects the body's resistance and response to treatment under these conditions (16). In particular, patients with gastric cancer are prone to poor nutritional status, which influences the occurrence of postoperative complications and the prognosis of patients undergoing surgery (3, 17). In this study, we investigated the relationship between nutritional status and short- and long-term prognoses.

When inflammation occurs in the body owing to malignancy, infection, or trauma, an acute-phase response is triggered to maintain homeostasis. The proteins that change in response to this process are known as acute-phase proteins. CRP is a protein produced by hepatocytes in response to stimulation by inflammatory cytokines such as vascular endothelial growth factor and interleukin-6 (IL-6). Albumin, also produced by hepatocytes, is regulated by inflammatory cytokines such as interleukin-1 (IL-1), IL-6, and tumor necrosis factor-alpha (TNF-α). In response to the exacerbation, progression, and metastasis of inflammation in cancer tissues, the CRP concentration increases, whereas that of albumin decreases (18). Patients with gastric cancer often exhibit elevated serum CRP levels. High serum CRP levels negatively affect prognosis, and a nutritional assessment index based on serum CRP levels has been correlated with postoperative complications and long-term prognosis in patients with gastric cancer (5, 19). Similarly, serum albumin levels, which are correlated with nutritional status, general condition, and disease progression, negatively affect the prognosis (6). Nutritional assessment indices based on serum albumin levels have been shown to correlate with prognosis and postoperative complications in patients with gastric cancer (4, 6, 20).

Lymphocytes play a central role in antitumor immunity by suppressing tumor cell growth and invasion through cytokine-mediated cytotoxicity (21), thus reflecting the functional impairment of adaptive cellular immunity against cancer cells. In patients with gastric cancer, the NLR, PLR, and CONUT score, which are nutritional indices based on lymphocyte counts, are associated with long-term prognosis and perioperative complications (7, 22–24).

Various nutritional indices have been used to evaluate the relationship between the nutritional status and prognosis. Among these newly established nutritional indices is the CI, which evaluates nutritional status based on three factors: serum albumin level as an indicator of nutritional status, lymphocyte count as an indicator of immune status, and CRP as an indicator of inflammatory status. Iida et al. reported that CI is a useful prognostic factor for hepatocellular carcinoma, and the relationship between CI and short- and long-term prognoses has also been reported for other carcinomas (11, 12). However, only a few reports have described the use of CI in the treatment of gastric cancer. Preoperative use of CI may help identify patients with impaired nutritional and immunological status and predict poor short- and long-term prognoses in these patients. Therefore, this study investigated the usefulness of CI as a prognostic factor for short- and long-term postoperative outcomes in patients with gastric cancer.

In Cohort 1 in the present study, patients with low CI were significantly more likely to have advanced gastric cancer and undergo open surgery and extensive lymph node dissection. Open surgery is often performed in patients with advanced clinical stages, and extensive lymph node dissection is required. Moreover, the more invasive the surgery, the higher the incidence of postoperative complications. Therefore, we applied propensity score matching to adjust for clinical stage, surgical approach, and extent of lymph node dissection between the two groups. It should be noted that, despite propensity score matching, BMI remained imbalanced with an SMD exceeding 0.5. This imbalance likely reflects the clinical characteristics of the low-CI group, as low BMI is inherently associated with poor nutritional status and a low CALLY Index. Additionally, gender and histological type showed SMDs exceeding 0.2. However, these factors are considered to have a minimal impact on surgical outcomes, as they are primarily associated with oncological prognosis rather than postoperative complications. In addition, the cN factor showed an SMD of 0.10, suggesting a negligible imbalance, while the extent of lymph node dissection was balanced between the groups, minimizing its potential influence on surgical invasiveness and postoperative complication rates. Therefore, the residual imbalances in these variables are unlikely to affect the interpretation of postoperative complication rates in this study. The postoperative complication rate of C-D Grade II or higher was 18.3% for all patients before matching and increased to 35.0% in the matched patients. This increase may be attributed to the higher proportion of patients with advanced gastric cancer in the matched group, which resulted in a more invasive surgical approach with open laparotomy and a higher proportion of D2 lymph node dissection.

Compared with the high-CI group, the low-CI group had a significantly higher incidence of postoperative complications and longer hospital stay. Additionally, a detailed examination of postoperative complications revealed no significant differences in intra-abdominal infectious complications, such as pancreatic fistula, intra-abdominal abscess, and anastomotic leakage; however, the incidence of postoperative pneumonia was significantly higher in the low-CI group. This finding can be attributed to the heightened vulnerability of the respiratory system to immunosuppression. The respiratory system is more susceptible to the effects of immune dysfunction. Postoperative immunosuppression and systemic inflammatory responses are well-known to increase the risk of respiratory infections. In contrast, intra-abdominal complications primarily result from surgical factors. While immune dysfunction can contribute as a secondary factor, exacerbating infections or delaying wound healing, the primary determinants of intra-abdominal complications are generally related to the extent of surgical invasion. Therefore, compared with intra-abdominal complications, the respiratory system is more likely to be affected by postoperative immune suppression, which may explain the relatively higher incidence of pneumonia observed in the low-CI group.

Several factors may explain the high incidence of postoperative complications in patients with low CI scores. First, systemic inflammation and poor nutritional status may compromise the immune system, leading to increased risk of postoperative complications, increased susceptibility to infection, and delayed wound healing (25). Furthermore, cancers in the low-CI group may have demonstrated greater invasiveness, necessitating open surgery or extensive lymph node dissection. Laparoscopic surgery for advanced gastric cancer has a better short-term prognosis and non-inferior long-term outcome than open surgery (26). Therefore, minimally invasive surgery for advanced gastric cancer may reduce the incidence of postoperative complications. Furthermore, respiratory rehabilitation and nutritional therapy may have been particularly effective in the low-CI group.

Cohort 2 showed no significant differences in sex between the high- and low-CI groups; however, the low-CI group had a higher proportion of older patients, elevated serum CEA levels, higher number of cases that underwent open surgery, greater incidence of postoperative complications, and more advanced pathological stages. Long-term prognosis was also significantly worse in the low-CI group. Multivariate analysis of prognostic factors for OS revealed that age, surgical approach, venous invasion, and CI were independent prognostic factors, with the hazard ratio of CI being higher than that of any other factor. CI is a prognostic factor for OS in ovarian and oral cancers; thus, the results of the present study are consistent with those of other studies (27, 28). Similarly, the analysis of prognostic factors for RFS revealed that age, venous invasion, and CI were major independent factors. Notably, although CI was an independent prognostic factor for OS and RFS, its impact was most pronounced for OS, for which it demonstrated the highest HR among all evaluated factors. For RFS, its influence remained significant, though not as dominant as for OS.

In an analysis focusing on patients with pStage I disease, the 5-year OS rate was 87.9% (95% confidence interval: 64.1–100.0%). A significant difference was observed between the high-CI and low-CI groups, with the low-CI group showing a poorer 5-year OS rate (69.1%, 95% confidence interval: 48.3–89.9% vs. 90.5%, 95% confidence interval: 71.8–100.0%; *p* = 0.0051). The 5-year RFS rate also demonstrated a significant difference, with the low-CI group having a poorer 5-year RFS rate compared to the high-CI group (69.1%, 95% confidence interval: 48.3–89.9% vs. 89.9%, 95% confidence interval: 84.9–94.9%; *p* = 0.0071). These results suggest that CI correlates with OS and RFS because it reflects the patients' systemic inflammation and nutritional status, encompassing a range of systemic conditions, including non-cancer health risks. These findings further support the notion that CI reflects patients' overall nutritional status and general condition.

In terms of the DSS, there was no significant difference between the high-CI and low-CI groups in patients with pStages I–III disease (91.2%, 95% confidence interval: 84.9–92.9%, vs. 89.3%, 95% confidence interval: 81.5–100.0%, *p* = 0.61). This suggests that while CI strongly correlates with OS and RFS, its association with DSS is limited. The lack of a significant difference in DSS may indicate that CI predominantly reflects factors such as systemic inflammation or nutritional status, which affect OS more than cancer-specific mortality. Furthermore, this finding suggests that non-cancer-related factors play a substantial role in the poorer OS observed in low-CI patients, emphasizing the need for comprehensive management of systemic health beyond cancer treatment.

Low-CI patients with pStages II/III disease were significantly less likely to receive adjuvant chemotherapy. The poorer 5-year OS and RFS rates in the low-CI group could possibly be explained by the higher prevalence of advanced gastric cancer, the lower likelihood of receiving adjuvant chemotherapy postoperatively, and their poor general condition after surgery.

To improve the long-term prognosis, future strategies should prioritize minimally invasive surgical techniques to reduce procedural invasiveness, while meticulously preventing postoperative complications. Avoiding complications is crucial to maintaining patients' condition and ensuring the feasibility of adjuvant chemotherapy during the postoperative period. The ability to administer adjuvant chemotherapy is particularly important for patients with low CI, as it significantly impacts overall outcomes.

CI offers three significant clinical advantages. First, it is easy to calculate and readily applicable in clinical practice. Second, the preoperative CI is a reliable predictor of postoperative complications, particularly postoperative pneumonia, and can be used to identify patients requiring close monitoring and early intervention. Third, CI is a valuable predictor of long-term survival, facilitating the selection of patients who could benefit from nutritional therapy to improve their overall condition. The cutoff values of CI in this study were different from those reported previously; however, the optimal cutoff value is likely to differ among various carcinomas (28). The cutoff value of the CI observed in this study differed from those previously reported. In Iida et al.'s study, the cutoff value for the CI in HCC patients was set at 5, whereas in our study, it was determined to be 2. This discrepancy may be attributed to a comparative analysis of the laboratory data from patients included in both studies. Specifically, the median serum CRP level in our Cohort 1 was 0.06 mg/dL, which was ~0.6 times lower than the 0.10 mg/dL reported for HCC patients in Iida et al.'s study. In contrast, there were no significant differences in the serum albumin levels (4.2 vs. 4.2 g/dL) and lymphocyte counts (1,600 vs. 1,400/μL). This lower CRP level could be attributed to the lower cutoff value of CI in our study. Therefore, a cutoff value of 2 was established to reflect the distinct inflammatory status of our gastric cancer cohort, providing a more appropriate and meaningful prognostic threshold for risk stratification (10).

The prognostic significance of the CI may extend beyond its individual components, reflecting an intricate interplay between systemic inflammation, nutritional status, and immune competence. This interplay shapes the tumor microenvironment, influences immune surveillance, and alters metabolic homeostasis, all of which contribute to the progression of gastric cancer and patient outcomes. From a clinical perspective, the CI offers significant practical value as a simple yet comprehensive preoperative assessment tool. A lower CI is associated with an increased risk of postoperative complications, particularly infectious events such as pneumonia, and is linked to poorer long-term outcomes, including overall survival (OS) and recurrence-free survival (RFS). This makes CI particularly useful for stratifying patients by surgical risk and guiding personalized perioperative management strategies. For instance, patients with low CI may benefit from preoperative nutritional support, immune-enhancing interventions, and the prioritization of minimally invasive surgical techniques to reduce surgical stress and complication rates. Additionally, CI can help identify patients who may require more intensive postoperative monitoring and early initiation of adjuvant therapies to improve long-term outcomes.

This study had some limitations. First, the small sample size might have decreased the power of detection. Excluding patients with more advanced disease (total gastrectomy, stage IV disease, cases with NAC) might have reduced the number of low CALLY index cases. Second, the retrospective, single-institutional nature of Cohort 2 could introduce case selection bias, limiting the external validity and generalizability of the results. Therefore, external validation using independent datasets from multiple centers is warranted to confirm the robustness and applicability of these findings across diverse patient populations and clinical settings. Nevertheless, despite these limitations, the CALLY index demonstrates significant potential as a practical and reliable predictive tool for postoperative outcomes in gastric cancer surgery, warranting further investigation through larger, multicenter prospective studies.

## 5 Conclusion

The CI is a valuable indicator of patients' overall conditions, providing predictive insights into short-term outcomes following gastric cancer surgery and long-term prognosis. Its ease in clinical practice can help guide treatment decisions and improve patient management.

## Data Availability

The original contributions presented in the study are included in the article/supplementary material, further inquiries can be directed to the corresponding author.
